# Validity of load rate estimation using accelerometers during physical activity on an anti-gravity treadmill

**DOI:** 10.1177/2055668320929551

**Published:** 2021-06-02

**Authors:** Susan Nazirizadeh, Maria Stokes, Nigel K Arden, Alexander IJ Forrester

**Affiliations:** 1 Faculty of Engineering & Physical Sciences, University of Southampton, Southampton, UK; 2 Faculty of Health Sciences, University of Southampton, Southampton, UK; 3 Centre for Sport, Exercise and Osteoarthritis Research Versus Arthritis, Southampton, UK; 4 MRC Lifecourse Epidemiology Unit, University of Southampton, Southampton, UK

**Keywords:** Physical activity monitoring, smartphone, smartwatch, linear mixed model, load rate monitoring, bootstrapping

## Abstract

**Introduction:**

A simple tool to estimate loading on the lower limb joints outside a laboratory may be useful for people who suffer from degenerative joint disease. Here, the accelerometers on board of wearables (smartwatch, smartphone) were used to estimate the load rate on the lower limbs and were compared to data from a treadmill force plate. The aim was to assess the validity of wearables to estimate load rate transmitted through the joints.

**Methods:**

Twelve healthy participants (female *n* = 4, male *n* = 8; aged 26 ± 3 years; height: 175 ± 15 cm; body mass: 71 ± 9 kg) carried wearables, while performing locomotive activities on an anti-gravity treadmill with an integrated force plate. Acceleration data from the wearables and force plate data were used to estimate the load rate. The treadmill enabled 7680 data points to be obtained, allowing a good estimate of uncertainty to be examined. A linear regression model and cross-validation with 1000 bootstrap resamples were used to assess the validation.

**Results:**

Significant correlation was found between load rate from the force plate and wearables (smartphone: $R^{2}=0.71$ ; smartwatch: $R^{2}=0.67$ ).

**Conclusion:**

Wearables’ accelerometers can estimate load rate, and the good correlation with force plate data supports their use as a surrogate when assessing lower limb joint loading in field environments.

## Introduction

### Background

Physical activity monitoring with inertial sensors is a growing field of research, with applications in elite sport,^1,2^ clinical conditions^3,4^ and the general population. Commercially available inertial sensors allow individuals to count steps, measure distances travelled and record physical activity duration, all of which may positively affect physical activity behaviour.^5^ However, excessive mechanical loading might be a risk factor for the progression of degenerative joint diseases such as osteoarthritis.^6^ Estimating (and so enabling the monitoring/control of) the loading on joints during physical activity in everyday life with commercially available inertial sensors may benefit some populations such as people with a high risk of developing degenerative joint diseases or people with arthritis.

The term ‘load’ describes biomechanical physical stresses which act on the body or anatomical structures within the body.^7^ These stresses can be kinetic, kinematic, oscillatory or thermal energy sources. In the present study, since kinetic energy sources are of interest, the term ‘load’ is strictly applied to weight-bearing forces on the joints. Load rate is the time derivative of this load.

### Load rate and its effects

While we are concerned with monitoring loading on joints during everyday life, much of the literature is in sports science, where repetitive loading on the lower limb joints is known to be a key component in the pathophysiology of stress fractures.^8^ Tibial stress fractures are related to tibial acceleration and vertical load rates.^8^ Daoud et al.^9^ showed on a large group of runners that higher positive vertical load rates were found mostly in people with tibial stress fractures in comparison to controls. These studies measured the load rate on the lower limbs with force plates, which are considered the gold standard for load rate measuring in biomechanical laboratories. However, being conducted in the biomechanical laboratory means that their methods are not suited for measuring the load rate of everyday activities. Pressure-measuring insoles are a valid and reliable method to measure ground reaction force without a force plate.^10,11^ However, due to their expense and cumbersome (often wired) nature, these too are unsuitable for taking measurements during everyday life. Development of a simple, portable and inexpensive method to quantify load rate on the lower limb joints during daily living is required.

### Estimating load rate using accelerometers

Commercially available acceleration sensors are commonly used for physical activity monitoring during everyday life.^12,13^ Neugebauer et al.^13^ developed a method for estimating peak vertical and braking ground reaction forces with accelerometers which they then validated against a force plate. The errors that were obtained are for peak vertical ground reaction forces (8.3%) and braking ground reaction forces (17.8%).

Some authors^12,13^ validated the use of accelerometers as tools for estimating peak ground reaction forces on force plates, with both studies yielding high correlation coefficient values. However, the focus of their validation was the peak ground reaction forces and not the load rate. The present study hypothesizes that load rates might be a better indicator for impact loading on the lower limb joints (following the literature^8,9^). Nevertheless, it should be mentioned that there may be other indications for joint damage such as biomechanics, age, strength, sex or predisposing conditions,^6^ which are not included in this paper.

Other features used for identifying impact loading can be found in elite sports research. Hollville et al.^14^ validated the MinimaxX accelerometer against a force plate by calculating the mean acceleration rate magnitude of the accelerometer and force plate (specific to a team sport activity performed on the force plate). The correlations between the accelerometer data and the force plate data were between 0.74 and 0.93. Their study supports the use of acceleration rate magnitude as a suitable method for capturing impact loadings on the lower limb joints. Wundersitz et al.^15^ assessed the validity of a MinimaxX accelerometer worn on the upper body for estimating peak forces during running and change-of-direction tasks. Peak vertical acceleration and acceleration magnitude values (m/s^2^) were converted to force values (N) via Newton’s second law of motion (i.e. multiplying by the participant’s body mass) and were compared against the peak ground reaction force from the force plate. They showed that accelerometers worn on the upper body could provide a relative measure of peak impact force experienced during running and two change-of-direction tasks (45° and 90°). This approach involved including the participant’s body mass in the equation, which was one of the hidden variables that Hollville et al.^14^ did not use. Since the accelerometer was attached to the upper body of the individuals, the actual accelerometer measurements came from the upper body, where a lighter/attenuated force was applied. This could be construed as not being an accurate way of measuring load. Nevertheless, as an estimation, it had high correlation with the ground reaction force and, hence, might be seen as a valid method for estimating ground reaction force with accelerometers.

Some authors^14,15^ validated two different acceleration values against force plate data: the mean acceleration rate (jerk) magnitude and the peak force (peak acceleration multiplied by the participant’s body mass). The approach in the present study is a combination of both quantities: the accelerometer rate magnitude was multiplied by the participants’ body mass to obtain an estimation of the load rate.

Although previous studies using accelerometers for the purpose of estimating ground reaction forces or accelerometer rates showed good correlations with respect to force plate data,^12–15^ validation studies assessing the relationship between load rate estimated with wearables and force plates are still necessary.

The aim of the current study was to assess the validity of load rate estimated with wearables against the ‘gold standard’ equipment, the force plate, during locomotive activities (walking, jogging, running) on an anti-gravity treadmill.

## Methods

The study design was cross-sectional. Twelve healthy adults (female *n* = 4, male *n* = 8; aged 26 ± 3 years; height: 175 ± 15 cm; body mass: 71 ± 9 kg; mean ± standard deviation) participated in the study. Participants were recruited via posters on multiple noticeboards around the University of Southampton. Once a participant showed interest, the researchers sent an email to them with the participant information sheet and an invitation to the study. Based on the screening, which excluded those with lower limb pathologies or any musculoskeletal, neurological or systemic diseases or other physical disabilities which may have limited their mobility, 12 of 18 volunteers accepted the invitation. Data collection took place at Southampton Football Club’s training facilities. The sample of convenience of 12 participants was chosen due to limited time and access to the facility. Each participant completed 18 different trials (six different bodyweight conditions: 30, 60, 80, 90, 100 and 110% × three speed conditions: 5, 8 and 12 km/h). The study was approved by the Faculty of Health Science Ethics Committee at the University of Southampton (no. 17086).

### Data collection

A simple Android app was used to acquire acceleration values from the microelectromechanical systems (MEMS) sensors in one smartphone between the shoulder blades (Smartphone 1, SP1) and one smartwatch on the right wrist (Smartwatch 1, SW1). All participants were asked to put on an elastic sports vest holding Smartphone 1, which was positioned in such a way as to have it located between their shoulder blades. This location aligns with elite sports practice,^1,2^ where athletes wear accelerometers between their shoulder blades. However, to simulate the real-world activity monitoring, an additional smartphone (Smartphone 2, SP2) was attached to the lateral right thigh with cohesive tape, and a smartwatch (Smartwatch 2, SW2) was placed on the left wrist. For Smartphone 2 and Smartwatch 2, only data for six participants were available due to technical limitations. The lateral right thigh was chosen to represent the usual position on the body of the smartphone: the hip pocket. The smartphones were Sony® Xperia™ Z Compact ( 127×65×9.5 mm, 137 g) and the smartwatches were Moto 360 from Motorola® ( 46×46×11 mm, 54 g).

Although the main objective of the study was to assess the validity of the wearables with respect to data from the treadmill force plate, the acceleration data from the wearables was also validated. Similar accelerometers were calibrated in previous works by Bassett et al.^16^ and Lee^17^ although only energy expenditure results were reported, while Boyd et al.^18^ assessed the validity of MinimaxX accelerometers for measuring physical activity in Australian football. The smartphone was mounted on a shaker (Brüel & Kjær (B&K) type 4809), with the smartwatch (with strap removed) and a B&K Type 4524-B lightweight triaxial piezoelectric OrthoShear accelerometer attached to the back of the phone via beeswax and tape. Data captured from all three devices during a 0–10 Hz sine sweep was aligned and resampled at 50 Hz. This frequency range resulted in a load rate range equivalent to that seen during the treadmill experiments ( $0<\left|\hat{\frac{\Delta F_{L}}{\Delta t}}\right|<4\times 10^{4}$ N/s), using *m* = 71 kg (see equation (2) below). The time domain root-mean-squared error ratios between the B&K accelerometer and smartphone and B&K accelerometer and smartwatch-derived load rate were 5.41% and 5.35%, respectively (*R*^2^ between all three devices was 1.00). These errors are 25.8% and 25.5% of the RMSER values for linear regression Model 1, detailed in ‘Linear regression models’ section. Measurement errors due to the sensors are therefore significantly smaller than other factors in the experiment.

The anti-gravity treadmill was the M320 from Alter-G®. The floor of the anti-gravity treadmill is mounted on four load cells which serve as a force plate. The voltage signals from the four load cells were collected with a sampling frequency of 128 Hz using four analogue inputs on a NI® DAQ USB™ device. The ported signal was collected with the LabVIEW™ software with the help of the data acquisition assistant. For the Alter-G anti-gravity treadmill, 128 Hz was the maximum sampling frequency available. Before the data collection, the force plate was calibrated with 25 weights between 0 and 90 kg. The weights used were weighed on a digital milligram scale and then placed in the centre of the force plate. The voltage signal for each weight was used for building a linear function ( $R^{2}=1.000$ ), which transformed voltage signal into force.

To test whether the wearables are able to estimate the amount of loading through joints, different joint loads needed to be tested, and the anti-gravity treadmill was one way of achieving this. It enabled the collection of multiple data points for varying speeds and gave a broad spectrum of different loading conditions on the joints. The treadmill comes with neoprene compression shorts that ensure an airtight seal in the enclosure. Air pressure lifts the participant off the treadmill floor, controlled by the weight measured by load cells beneath the floor. During the locomotive activities, the researcher changed randomly the bodyweight percentage (30, 60, 80, 90, 100 and 110%) setting, and the anti-gravity treadmill would lift the participants according to the percentage.

Another advantage of using the anti-gravity treadmill was that it has an integrated force plate, albeit with a sampling frequency somewhat lower than a biomechanics laboratory walk-way force plate (128  Hz vs. >500 Hz). The low sampling frequency of the wearables (50 Hz) required that we sampled many steps to obtain accurate data. However, a walk-way force plate would only allow one step to be recorded at a time. A treadmill was therefore more appropriate as a validation tool. The multiple steps recorded mitigate the reduced sampling frequency, with averaging over many steps used here instead of filtering a high frequency signal. Averaging over a large number of steps allows us to obtain a more accurate mean without smoothing problems from filtering.^12^

The speed conditions (5, 8 and 12 km/h) were chosen to obtain a broad locomotive range from walking, to jogging and then to running. Each trial lasted 90 s, with the smartphone, smartwatch and force plate data being collected simultaneously. The 90-s trials with a 60-s sampling window were a pragmatic balance between obtaining accurate mean values from the sensors and participant fatigue. Any possible effects due to fatigue were further mitigated by allowing a rest period between trials. The first 20 s of recording served as a period of habituation and were discarded before the data were processed. The next 60 s were used for data processing, while the last 10 s of each trial were discarded to avoid recording possible behaviour changes associated with the trial ending.

### Data processing

The data were processed using MATLAB (Version R2016b, The Math Works®, Natick, MA).

If the infinitesimal calculus of the load rate is defined as

(1) $${\dot{F}}_{L}=\frac{dF_{L}}{dt}=m\;\frac{da}{dt},$$

the estimated mean load rate magnitude is

(2) $$\bar{\left|\hat{\frac{\Delta F_{L}}{\Delta t}}\right|}=\frac{1}{n-1}\sum_{j=1}^{n-1}m\;\sqrt{\sum_{i=1}^{3}{\left(\frac{a_{i,t_{j+1}}-a_{i,t_{j}}}{\Delta t}\right)}^{2}}$$

where *a*_1_, *a*_2_, *a*_3_ are the acceleration in the *x*, *y*, *z* directions and *n* the number of data samples at interval Δt . With units of kg m/s^3^ = N/s, this estimated mean load rate magnitude was used for the remaining analyses (m = meter, s = seconds, N = newton and kg = kilogram).

### Linear regression models

A linear mixed regression model was chosen due to the existence of hidden variables which were not measured while collecting the data, such as anatomy, muscle strength and the style of the gait of the individuals. The load rate data from the force plate was the response variable, the data from the wearables, the predictor variables and the participants were all the grouping variable.

The data were used to build three different linear regression models.

Model 1 (M1)

(3) $$y_{m,i}^{M1}=︸\underset{\text{Fixed effect}}{\alpha_{\mathrm{Wear}}+\beta_{\mathrm{Wear}}x_{m,i}}$$

is a linear model with fixed effects, considering only the population’s average behaviour and ignoring the between-subject variation in ambulatory activities.

Model 2 (M2)

(4) $$y_{m,i}^{M2}=︸\underset{\text{Fixed effect}}{\alpha_{\mathrm{Wear}}+\beta_{\mathrm{Wear}}x_{m,i}}+︸\underset{\text{Random effect}}{a_{i}}$$

is a linear mixed model with random intercept, which assumes that the between-subject variation affects only this random intercept.

Model 3 (M3)

(5) $$y_{m,i}^{M3}=︸\underset{\text{Fixed effect}}{\alpha_{\mathrm{Wear}}+\beta_{\mathrm{Wear}}x_{m,i}}+︸\underset{\text{Random effect}}{a_{i}+b_{i}x_{m,i}}$$

is a linear mixed model with random intercept and slope, allowing for the between-subject variation affecting both the intercept and slope.

The estimated mean load rate magnitude (2) from the force plate (the response variable) is $y_{m,i}$ for observation *m* and participant *i*, $\alpha_{\mathrm{Wear}}$ and $\beta_{\mathrm{Wear}}$ are the intercept and slope of the estimated load rate of the wearables (fixed effect predictor variables) and $a_{i}$ and $b_{i}$ are the intercept and slope of each participant (random effect predictor variables).

To obtain a better indication of uncertainty in our models, the bootstrapping resampling method was used, wherein vectors of the same sample length as the original data are created by drawing, with replacement, random observations from the original data set.^19^ One thousand bootstrap vectors were created and cross-validated.^20^ For every vector, three models were built: Model 1, Model 2 and Model 3. For the three models, the *R*^2^ and root-mean-squared error ratios ( $\mathrm{RMSER}=\frac{\sqrt{\mathrm{MSE}}}{{\bar{y}}_{\text{forceplate}}}$ ) were calculated. Confidence intervals were based on the 1000 bootstrap samples. Cross-validation has the advantage that it provides a direct estimate of test errors.

### Statistical analysis

A one-way ANOVA was used to determine if there was a significant difference between the mean of the bootstrapped *R*^2^ and RMSER values of Models 1, 2 and 3 followed by a pairwise comparison with the Bonferroni correction.^21^ The Bonferroni correction was used to include the effect of comparing multiple groups. Hence, the desired p-value has to be divided by the number of comparisons being conducted, and so a value of α=0.05/4=0.0125 was used for significance. The *R*^2^ and RMSER values of each model were normally distributed (*p* > 0.15). One-way ANOVA with the Bonferroni correction was used to compare the *R*^2^ values of the four different devices (Smartphone 1, Smartwatch 1, Smartphone 2, Smartwatch 2). For comparing the devices with each other, α=0.05/6=0.0083 was used for significance.

## Results

Participants 1–11 completed all percentage bodyweight trials at the three speeds mentioned above. Participant 12, however, was only able to complete the 5 and 8 km/h trials due to time restrictions. Furthermore, the complete data from participant 1 and the 5 km/h data from participant 2 were identified as outliers and were removed. Therefore, a total of 186 trials were analysed.

**Figure 1.**
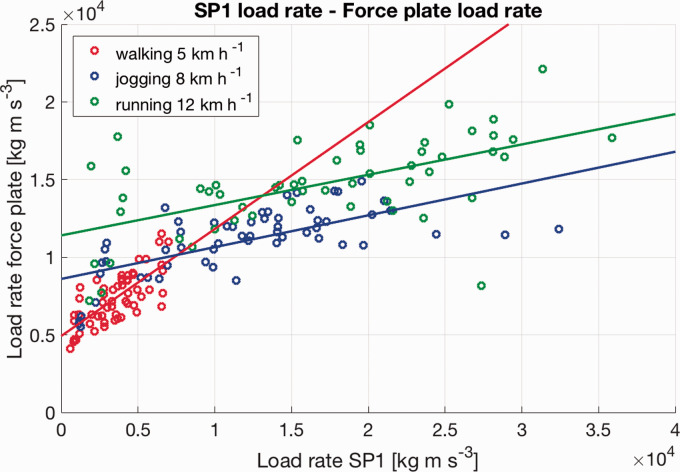
Whole dataset of all participants for Smartphone 1 with linear regression lines.

**Figure 2.**
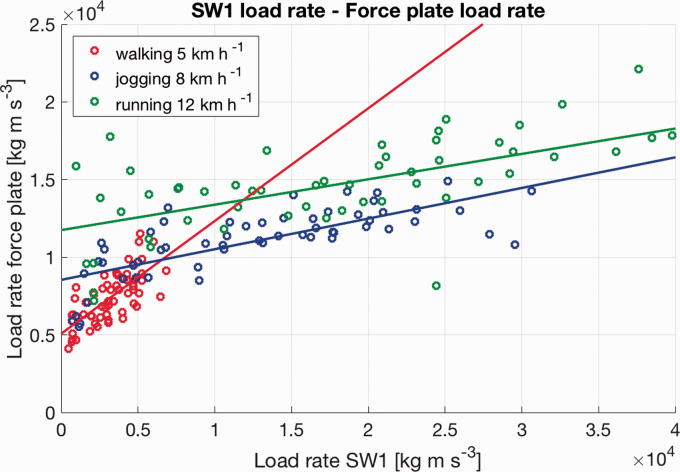
Whole dataset of all participants for Smartwatch 1 with linear regression lines.

The linear relationship between load rates from the wearables and the load rates from the force plate can be seen in Figures 1 and 2. The plots show all data points of Smartphone 1 and Smartwatch 1 with linear regression lines.

For both the *R*^2^ and RMSER values, 95% confidence intervals were calculated (Table 1). The *R*^2^ values of the three models for Smartphone 1 are $R_{M1}^{2}=0.60_{0.48}^{0.71},\;R_{M2}^{2}=0.68_{0.54}^{0.80}$ and $R_{M3}^{2}=0.71_{0.60}^{0.81}$ , demonstrating a linear relationship exists for all models between wearables and the force plate.

**Table 1.** $R_{\mathrm{Model}}^{2}$ and RMSER values, ± 95% confidence intervals for all participants using all of the smartphone and smartwatch data which was collected.

Device	Model 1	Model 2	Model 3
SP1 – *R*^2^	$0.60_{0.48}^{0.71}$	$0.68_{0.54}^{0.80}$	$0.71_{0.60}^{0.81}$
SP1 – RMSER	$0.21_{0.18}^{0.24}$	$0.19_{0.15}^{0.22}$	$0.18_{0.15}^{0.21}$
SW1 – *R*^2^	$0.60_{0.48}^{0.70}$	$0.63_{0.49}^{0.74}$	$0.67_{0.55}^{0.78}$
SW1 – RMSER	$0.21_{0.19}^{0.24}$	$0.20_{0.17}^{0.24}$	$0.19_{0.16}^{0.22}$

The one-way ANOVA showed that the three models had a significant difference (*p* < 0.0001). The pairwise comparison showed that Model 3 is the best choice.

For Model 3, Smartphone 1, the performances of the models for the three different speed conditions are $R_{5km/h}^{2}=0.86_{0.76}^{0.93},\;R_{8km/h}^{2}=0.74_{0.52}^{0.90}$ , and $R_{12km/h}^{2}=0.77_{0.56}^{0.90}$ (see Table 2).

**Table 2.** $R_{\mathrm{speed}}^{2}$ values, ±95% confidence intervals for each speed for Smartphone 1 (between the shoulder blades).

Speed (km/h)	Model 1	Model 2	Model 3
5	$0.51_{0.29}^{0.69}$	$0.83_{0.72}^{0.90}$	$0.86_{0.76}^{0.93}$
8	$0.40_{0.21}^{0.62}$	$0.69_{0.53}^{0.83}$	$0.74_{0.52}^{0.90}$
12	$0.28_{0.02}^{0.54}$	$0.47_{0.13}^{0.77}$	$0.77_{0.56}^{0.90}$

All differences in the models were significant (*p* < 0.0001, α=0.05/4=0.0125 ).

The *R*^2^ values of the four devices were (all for Model 3): $R_{SP1}^{2}=0.79_{0.69}^{0.87},\;R_{SP2}^{2}=0.78_{0.66}^{0.87},\;R_{SW1}^{2}=0.75_{0.62}^{0.86},\;R_{SW2}^{2}=0.77_{0.66}^{0.87}$ (Table 3). The *R*^2^ values of each model and device were normally distributed (Kolmogorov–Smirnov, $p_{5km/h}=0.89,\;p_{8km/h}=0.28,\;p_{12km/h}=0.81$ ).

**Table 3.** $R_{\mathrm{speed}}^{2}$ values, ±95% confidence intervals for the devices at different body locations (for six participants).

Device (location)	Model 1	Model 2	Model 3
SP1 (between shoulder blades)	$0.65_{0.51}^{0.77}$	$0.76_{0.65}^{0.86}$	$0.79_{0.69}^{0.87}$
SW1 (right wrist)	$0.64_{0.52}^{0.75}$	$0.70_{0.56}^{0.80}$	$0.75_{0.62}^{0.86}$
SP2 (right hip)	$0.75_{0.62}^{0.85}$	$0.77_{0.62}^{0.87}$	$0.78_{0.66}^{0.87}$
SW2 (left wrist)	$0.69_{0.56}^{0.80}$	$0.75_{0.62}^{0.84}$	$0.77_{0.66}^{0.87}$

Smartwatch 1 had a significant different mean (*p* < 0.0001, α=0.05/4=0.0125 ).

The one-way ANOVA showed that there was a significant difference (*p* < 0.0001). And the pairwise comparison showed that just Smartwatch 1 was significantly different (*p* < 0.0001, α=0.05/6=0.0083 ).

The data for this current study are available on Github.^22^

## Discussion

The present findings show *R*^2^-values between 0.28 and 0.86 for force plate and wearable estimates of load rate data, while the participants performed locomotive activities on an anti-gravity treadmill. In this section, the different models (Model 1, Model 2, Model 3), the models with different speed conditions (5, 8 and 12 km/h), and the difference between the wearables on different body parts are discussed.

In Figures 1 and 2, the linear regression lines for walking have a higher slope than the slopes of the jogging or running data. Looking in detail at the slope of the walking data, the wearables seem to underestimate the load rate in comparison to the force plate. This indicates that wearables might slightly underestimate the load rate for low-intensity activities. For jogging and running, however, it seems that the wearables mostly overestimated the load rate data in comparison to the force plate data. This speed-dependent relationship highlights that, although data from wearables might be used as a surrogate for ground reaction data, it is not a direct replacement. This information is important if future applications are being developed. For each activity, a dedicated model might lead to better predictions.

To assess the validation of load rate estimated with wearables against the force plate during locomotive activities, two linear mixed regression models and a linear regression model were developed. A one-way ANOVA showed that all models were significantly different from each other (*p* < 0.0001). The pairwise comparison helped to identify the best model, which was Model 3. The difference between Model 1 and Model 3 was the highest with $\Delta R_{M3,M1}^{2}=0.11,\Delta \mathrm{RMSE}R_{M3,M1}=-0.031$ . Hence, knowing that Model 3 had the highest *R*^2^ and lowest RMSER values would lead to the decision that Model 3 ( $R_{M3}^{2}=0.71_{0.60}^{0.81}$ ) is the best performing model. Model 3 included, in comparison to Model 1, random slope and intercept effects, which takes into account unknown participant-specific characteristics, such as muscle structure, skeletal structure or participant height, all of which are hidden variables for the model. To examine a simpler model, the random slope of Model 3 was excluded: i.e. Model 2 with a fixed effect and a random intercept, which led to a lower $R_{M2}^{2}=0.68_{0.54}^{0.80}$ . Therefore, Model 2 implies that different participants did, indeed, have hidden variables which, in turn, influenced the slope and intercept of the function. Nevertheless, the improvement of Model 3 over Model 2 was small with $\Delta R_{M3,M2}^{2}=0.080$ .

It was essential to consider Model 1 ( $R_{M1}^{2}=0.60_{0.48}^{0.71}$ ), with just fixed effects, to be able to develop a baseline model. Adding random slope and intercept effects creates a more accurate model but with the disadvantage of being a less generalizable model. Neugebauer et al.^13^ also created linear mixed models for their analysis, which were in comparison to the models in this study much more complex. They considered the predictor variables: acceleration, participant mass, type of activity (walk = 0, run = 1) and interaction between acceleration data and type of activity. This complex model yielded a small absolute error value of 8.3%, where the type of activity had the most significance in the model. This led to the decision to conduct further analysis considering the speed condition (5, 8 and 12 km/h) to be able to compare the model from Neugebauer et al.^13^ with Model 3 in this study.

When comparing the different speed conditions recorded with Smartphone 1, it can be seen that for Model 3, the *R*^2^ values do not vary substantially ( $R_{5km/h}^{2}=0.86_{0.76}^{0.93},\;R_{8km/h}^{2}=0.74_{0.52}^{0.90}$ and $R_{12km/h}^{2}=0.77_{0.56}^{0.90}$ , Table 2). The *R*^2^ value for the 5 km/h, however, was the highest. This implies that the model was suited to monitoring people using wearables at varying speeds: e.g. covering the range of people with a slower gait to people with faster gaits. Knowing the speed of the locomotive activity increases the *R*^2^ and yields similar results to those of Neugebauer et al.^13^ However, Model 3 is less complex and has just one prediction variable (load rate estimated by wearables) and one grouping variable (participant), which leads to a direct relation between load rate estimated by wearables and load rate estimated by force plates.

When comparing the wearables (Smartphone 1, Smartwatch 1, Smartphone 2, Smartwatch 2) attached to different body parts, all devices had very similar *R*^2^ values ( $R_{SP1}^{2}=0.79_{0.69}^{0.87},\;R_{SP2}^{2}=0.78_{0.66}^{0.87},\;R_{SW1}^{2}=0.75_{0.62}^{0.86},\;R_{SW2}^{2}=0.77_{0.66}^{0.87}$ , Model 3 results, see Table 3). However, the pairwise comparison showed that Smartwatch 1 differed from the other three devices ( α=0.05/6=0.0083 ). Smartwatch 1 was on the right wrist, which most often deviated from a consistent motion (for actions such as stroking one’s hair, looking at the smartwatch or gesticulating). These results imply that the suitability of wearables as a surrogate for ground reaction load is largely independent of location on the body. However, the authors propose that for further research, the non-dominant wrist of the participant is used to avoid confounders. Also, it is suggested that between the shoulder blades and the right hip are suitable locations future studies.

The comparison between the three models may help other researchers understand the generalizability of the methods used in the present study. Neugebauer et al.^13^ used a complex generalized regression model, which included acceleration, weight, type of activity and the interaction between the type of activity and acceleration. The generalization of their model is difficult due to its complexity. The models used here are kept as simple as possible. Hence, the load rates estimated with the wearables and force plate are directly related to the models. Another finding was that knowing the speed of the activity increased the quality-of-fit. Considering the speed led to similar results to Neugebauer et al.^13^ which included the ‘type of locomotion (walk or run). However, including the speed in the model makes the model less general, hence, less useful for monitoring everyday living.

Another added complexity of the models of Meyer et al.^12^ and Neugebauer et al.^13^ is, unlike load rate estimation, the requirement for an algorithm to identify peak accelerations (which may also lead to errors when analysing noisy signals).

A major limitation of the four previous validation studies^12–15^ is that all force plates were placed in the middle of the laboratory, thereby giving the participants between 10 and 15 m to perform the activities. Except for Hollville et al.^14^ who used six force plates, all other studies used one force plate in the middle of the room. One force plate means that, for each trial, data for just one step was available. Hollville et al.^14^ and Wundersitz et al.^15^ repeated their trials around six to seven times to obtain a better estimate of the uncertainty. The force plate integrated treadmill, on the other hand, generated data for every step over the 60s sampling time. A better estimate of uncertainty in the data could therefore be made. Furthermore, with the treadmill, a period of habituation for 20 s of walking, jogging or running was possible during each trial, which would not have been possible if the participants just had 10–15 m in which to do the activities. Additionally, to obtain a better estimate of the uncertainties in the models, bootstrapping and cross-validation were used.

### Limitations

One of the weaknesses of this study was the limited number of participants. A larger number of participants would have been desirable but, due to restricted time at the facility, the number was kept to 12 participants.

The lower limb has in some sense been treated as a single segment, rather than a complex chain of joints, whose interactions might vary based on age, strength, gender or predisposing conditions.

Participants’ trainers (shoes) were not standardized, which could be a confounder as they have different absorption properties.

One limitation was the number of wearables attached to the participants. A greater number would have given a better understanding of the position of the wearables on the participants’ bodies and how they affect the load rate data.

Improved *R*^2^ and RMSER values might have been achieved with more participants and higher sampling rates (the on-board sensor sampling rates of wearables continue to improve with the development of the technology).

There are studies showing that a force measuring treadmill produces noise due to the treadmill.^23^ This was not included in the analysis and might be a limitation of the study.

## Conclusion

Smartphones appear to provide an acceptable level of accuracy for estimating load rate on the lower limbs during locomotive activities on a treadmill. The best model was Model 3 with 71% validity. The term ‘acceptable’ is warranted because the correlation found between load rate data from the wearables and the force plate can be described as a ‘high positive correlation’ from the guidelines of Hinkle et al.^24^ The present results may, therefore, be considered as positive. The models’ validity was high for varying speeds. Therefore, it is suitable for a range of activities from everyday to the athletic.

These positive results support further research in using wearables to estimate load rate, which may lead to a progressive development in healthcare and the self-management of arthritis and exercise. Wearables with load rate estimation may provide an easy, objective and cost-effective method for people to measure their activity concerning the load on their joints during daily activities.
